# Dictamnine Exhibits Anti-Asthmatic Effects by Modulating TGF-β/Smad2/3 Signaling in a Murine Asthma Model and Human Bronchial Epithelial Cells

**DOI:** 10.3390/ijms262411891

**Published:** 2025-12-10

**Authors:** Myung-A Jung, Bu-Yeo Kim, Joo Young Lee, Kon-Young Ji, Mi Han Lee, Dong Ho Jung, Mudan Cai, Taesoo Kim

**Affiliations:** 1 KM Convergence Research Division, Korean Institute of Oriental Medicine, Daejeon 34054, Republic of Korea; 2 Research Infrastructure Team, R&D Strategy Division, Korean Institute of Oriental Medicine, Daejeon 34054, Republic of Korea; 3 KM Science Research Division, Korean Institute of Oriental Medicine, Daejeon 34054, Republic of Korea

**Keywords:** dictamnine, allergic asthma, TGF-β/Smad2/3, anti-asthmatic, airway inflammation, epithelial integrity

## Abstract

Current asthma therapies reduce inflammation and symptoms but there are concerns regarding adverse effects and the long-term treatment burden. The anti-asthmatic potential of Dictamnine (Dic) has not been investigated. The therapeutic effect of Dic on airway inflammation and remodeling was investigated by targeting the tumor growth factor (TGF)-β/Smad2/3 pathway. A murine model of ovalbumin (OVA)-induced asthma was used to evaluate the effects of orally-administered Dic on airway hyperresponsiveness, inflammatory cytokines in bronchoalveolar lavage fluid (BALF), OVA-specific IgE in the serum, and histopathological changes. The expression of TGF-β/Smad2/3 and epithelial markers was assessed. Human bronchial epithelial cells were used in vitro to examine the effects of Dic on TGF-β-induced Smad2/3 phosphorylation. Network pharmacology was conducted to predict Dic-associated targets and pathways. Dic substantially reduced the levels of Th2 cytokines, mucin 5AC in BALF, and OVA-specific IgE in the serum. Histology indicated reduced inflammatory cell infiltration, bronchial wall thickening, and peribronchial fibrosis in Dic-treated mice. Dic downregulated TGF-β and p-Smad2/3 expression and upregulated ZO-1 expression in the lung tissue. Dic downregulated TGF-β-induced Smad2/3 phosphorylation in bronchial epithelial cells. Network pharmacology indicated enrichment of Dic-related genes in the TGF-β pathway. Dic exhibited anti-asthmatic effects and is a potential therapeutic candidate.

## 1. Introduction

Asthma affects approximately 3–4% of the global population, representing one of the most prevalent chronic respiratory diseases worldwide [1,2,3]. It is characterized by persistent airway inflammation, airway hyperresponsiveness (AHR), and airway remodeling [4]. AHR comprises both transient inflammation-driven and persistent remodeling-associated components, each contributing to airflow limitation and disease severity in responses to direct or indirect stimuli [5].

In asthma, immune cells (eosinophils, T-lymphocytes, and epithelial cells) release mediator-like cytokines and chemokines, driving disease progression [6]. Furthermore, the epithelial cells release profibrotic mediators, particularly TGF-β, which stimulates fibroblasts and myofibroblasts to produce collagen and other proteins, leading to airway wall thickening and remodeling. TGF-β regulates the inflammatory cascade and immune responses, making it a central player in airway remodeling and chronic asthma symptom development [6].

The TGF-β/Smad signaling pathway drives epithelial–mesenchymal transition (EMT) and airway remodeling [7]. The dual role of TGF-β as an inflammation mediator and driver of structural airway changes makes it a prime therapeutic target in asthma management [6].

In addition to inflammation, disruptions in epithelial barrier integrity are crucial in asthma pathogenesis. The airway epithelium acts as a physical and chemical barrier. Damage to this barrier facilitates the release of growth factors such as TGF-β [8]. Furthermore, goblet cell hyperplasia and excessive mucus production, regulated by IL-13 and mucin 5AC (MUC5AC), contribute to the exacerbation of airway narrowing and remodeling, thus worsening clinical symptoms [9].

Current asthma therapies effectively reduce inflammation and symptoms; however, concerns regarding adverse effect and long-term treatment burden remain [10,11,12]. Consequently, natural compounds are being explored as promising alternative therapeutic options.

Dictamnine, a fluoroquinolone alkaloid derived from *Dictamnus dasycarpus*, possesses anti-inflammatory and antioxidant properties [13]. A previous study demonstrated that *Dictamnus dasycarpus* extract attenuates airway inflammation and mucus hypersecretion in allergic asthma through the STAT6-STAT3/FOXA2 pathway [13], implying that its bioactive constituents, including dictamnine, may contribute to these pharmacological effects. Dictamnine scavenges reactive oxygen species, suppresses the activation of NLRP3 inflammasomes, and downregulates the expression of inflammatory cytokines, including IL-1β and TNF-α [14]. Additionally, dictamnine inhibits EMT in tumor models by modulating hypoxia-inducible factor (HIF-1α) and Slung expression, suggesting its broader effect on EMT-related pathways [15]. Because OVA-induced sensitization and challenge reproduce hallmark features of allergic asthma, OVA is widely used to establish experimental asthma models [16,17].

It is not clear whether dictamnine modulates asthma-related signaling pathways, particularly TGF-β/Smad2/3 signaling. Although dictamnine has shown promise in other inflammation models [18], its effects on asthma-specific features have not been elucidated. Therefore, in this study, we investigated the anti-asthmatic effect of dictamnine using OVA-induced murine asthma model and human bronchial epithelial cells.

## 2. Results

### 2.1. Dictamnine Mitigated AHR in Asthmatic Mice

**Figure 1:**
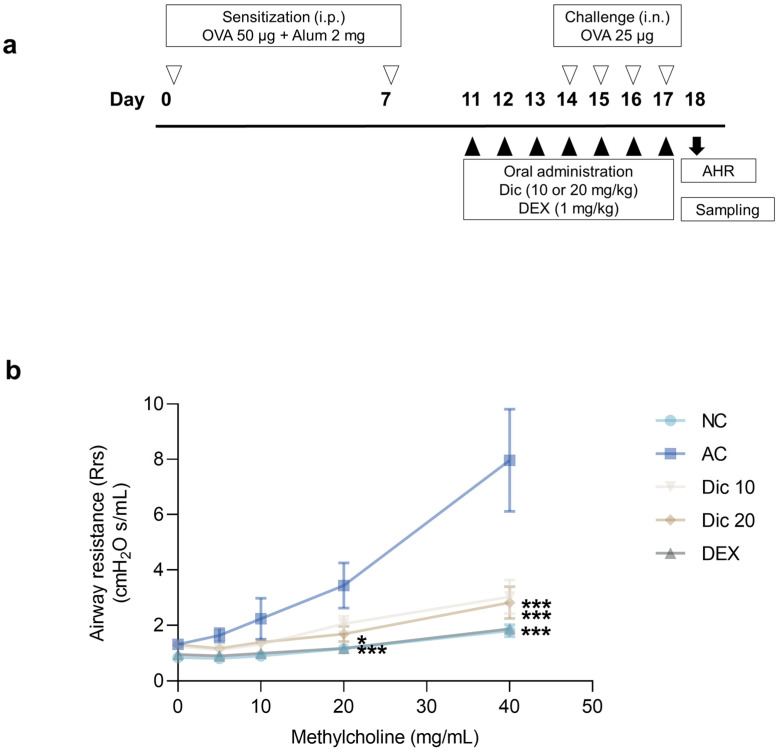
Experimental design and airway hyperresponsiveness (AHR). (**a**) Schematic of the OVA-induced asthma protocol and treatment schedule. i.p, intraperitoneal; i.n; intranasal. (**b**) Airway resistance (Rrs) was measured following methacholine challenges (5, 10, 20, and 40 mg/mL). Data are expressed as mean ± SEM (*n* = 4–5 per group). * *p* < 0.05, *** *p* < 0.001 compared to the AC group.

Airway resistance (Rrs) in the asthma control (AC) group was higher than that in the normal control (NC) group at both 20 mg/mL (NC: 1.15 ± 0.12 vs. AC: 2.88 ± 0.84) and 40 mg/mL (NC: 1.81 ± 0.24 vs. AC: 7.96 ± 2.41) methacholine concentrations (*p* < 0.001) (Figure 1b). Dictamnine treatment reduced Rrs compared with the AC group, at 20 mg/mL; only the Dic 20 group showed lower Rrs than the AC group (*p* < 0.05). At 40 mg/mL, both Dic 10 and Dic 20 groups showed lower Rrs values than the AC group (*p* < 0.001). The dexamethasone (DEX) group also showed markedly decreased Rrs at 20 and 40 mg/mL methacholine compared to the AC group (*p* < 0.001).

### 2.2. Dictamnine Alleviated Inflammatory Cell Infiltration in Asthmatic Mice

**Figure 2:**
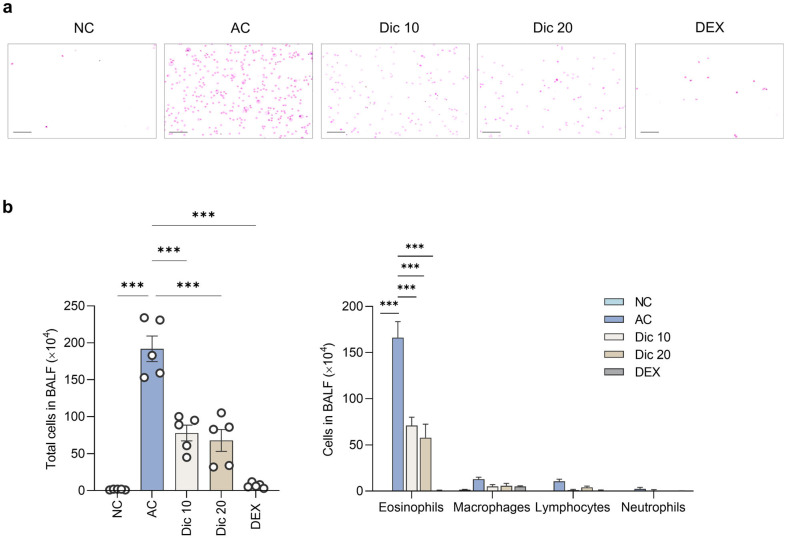
Dictamnine reduced inflammatory cell infiltration in OVA-induced asthmatic mice. (**a**) Representative Diff-Quik-stained images of BALF from NC, AC, Dic 10, Dic 20, and DEX groups. Scale bar = 100 μm. (**b**) Quantification of total (**left**) and differential (**right**) cell counts in BALF. Data are expressed as mean ± SEM (*n* = 5 per group). *** *p* < 0.001 compared to the AC group.

The OVA-challenged group (AC) showed higher total bronchoalveolar lavage fluid (BALF) cell counts (192.00 ± 17.29 × 10^4^) than the NC group (1.60 ± 0.24 × 10^4^) (*p* < 0.001) (Figure 2). However, both dictamnine-treated groups showed lower total BALF cell counts than the AC group (Dic 10: 78.00 ± 10.66 × 10^4^; Dic 20: 68.00 ± 14.78 × 10^4^, both *p* < 0.001).

The eosinophil counts in the AC group were higher than those in the NC group (AC: 166.20 ± 17.34 × 10^4^; NC: 0. *p* < 0.001). Dictamnine treatment decreased eosinophil counts (71.03 ± 8.84 × 10^4^; Dic 20: 57.87 ± 14.59 × 10^4^, both *p* < 0.001), but had no substantial effect on macrophage, lymphocyte, and neutrophil counts (*p* = NS).

### 2.3. Dictamnine Reduced Th2 Cytokine, MUC5AC, and OVA-Specific IgE Levels in Asthmatic Mice

**Figure 3:**
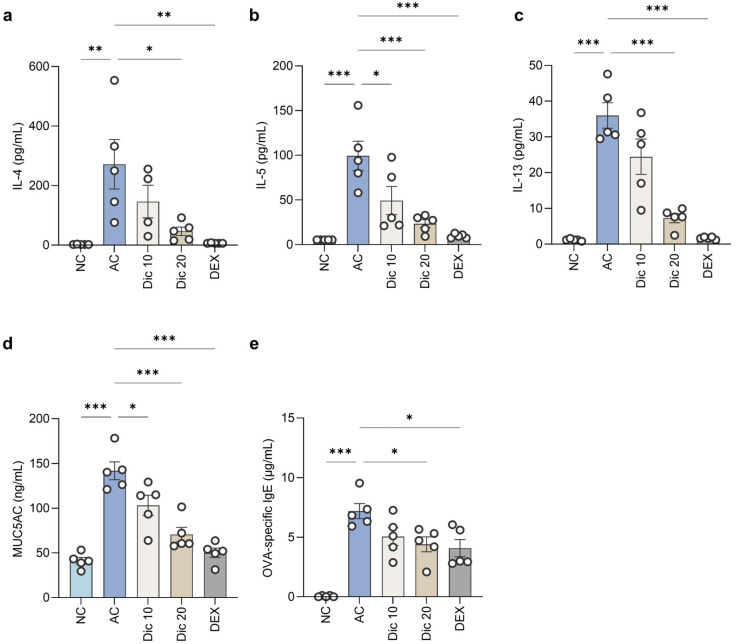
Dictamnine reduced Th2 cytokine, MUC5AC, and OVA-specific IgE levels in OVA-induced asthmatic mice. (**a**–**c**) BALF cytokine levels: (**a**) IL-4, (**b**) IL-5, and (**c**) IL-13. (**d**) MUC5AC levels in BALF. (**e**) OVA-specific IgE levels in serum. Data are expressed as mean ± SEM (*n* = 5 per group). * *p* < 0.05, ** *p* < 0.01, and *** *p* < 0.001 compared to the AC group.

Dictamnine treatment led to significant reductions in the levels of Th2 cytokines, MUC5AC in the BALF, and OVA-specific IgE in the serum (Figure 3). Dictamnine treatment at 10 and 20 mg/kg substantially reduced Th2 cytokine levels in the BALF. The IL-4 levels were markedly increased in the AC group (271.63 ± 82.98 pg/mL) compared to NC (2.58 ± 0.27 pg/mL). The Dic 20 group (46.54 ± 14.07 pg/mL) was lower than that in the AC group (*p* < 0.05). The IL-5 level was also elevated in AC (99.34 ± 16.41 pg/mL) compared to NC (5.40 ± 0.00 pg/mL). Both Dic 10 (49.28 ± 15.73 pg/mL, *p* < 0.05) and Dic 20 (23.49 ± 4.36 pg/mL, *p* < 0.001) had lower levels than the AC group. The IL-13 was higher in AC (36.00 ± 3.55 pg/mL) than in NC (1.20 ± 0.13 pg/mL). In contrast, Dic 20 (7.27 ± 1.27 pg/mL, *p* < 0.001) was lower than that in the AC group. Additionally, MUC5AC secretion was elevated in AC (141.84 ± 10.00 ng/mL) compared to NC (41.18 ± 3.82 ng/mL). Both Dic 10 (103.22 ± 11.40 ng/mL, *p* < 0.05) and Dic 20 (70.51 ± 8.14 ng/mL, *p* < 0.001) had lower MUC5AC levels compared to the AC group. Serum OVA-specific IgE levels were markedly increased in AC (7.20 ± 0.59 μg/mL) compared to the NC (0.05 ± 0.03 μg/mL), whereas both Dic 10 (5.06 ± 0.72 μg/mL, *p* < 0.05) and Dic 20 (4.20± 0.63 μg/mL, *p* < 0.05) exhibited lower OVA-specific IgE levels, indicating that dictamnine effectively mitigated allergic sensitization and inflammatory responses.

### 2.4. Dictamnine Attenuated Airway Inflammation and Hispathological Alterations in Asthmatic Mice

**Figure 4:**
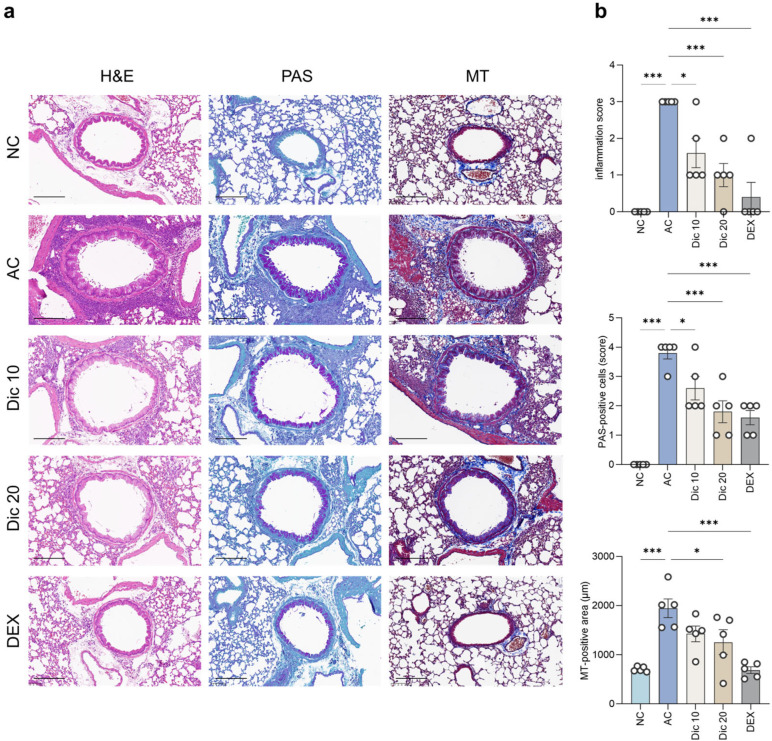
Dictamnine attenuated airway inflammation and fibrosis in OVA-induced asthma. (**a**) Representative images of H&E-, PAS-, and MT-stained lung sections from the NC, AC, Dic 10, Dic 20, and DEX groups. Scale bar = 100 μm. (**b**) Quantitative analysis of inflammation, PAS-positive goblet cells, and fibrotic area. Data are expressed as mean ± SEM (*n* = 5 per group). * *p* < 0.05 and *** *p* < 0.001 compared to the AC group.

In the NC group, the bronchial architecture remained intact, with no inflammatory infiltration (Figure 4). In contrast, the AC group (3.00 ± 0.00) showed marked inflammatory cell infiltration and substantial bronchial wall thickening. Both dictamnine-treated groups showed reduced inflammatory infiltration, with Dic 20 exhibiting pronounced improvement (1.00 ± 0.32, *p* < 0.001).

Periodic acid–Schiff (PAS) staining indicated an increase in PAS-positive goblet cells in the AC group (3.80 ± 0.20, *p* < 0.001), compared to NC (0.00 ± 0.00) indicating mucus hypersecretion (*p* < 0.001). Dictamnine treatment decreased PAS-positive goblet cell scores, with Dic 10 (2.60 ± 0.40, *p* < 0.05) and Dic 20 (1.80 ± 0.37, *p* < 0.001) both showing reduced scores compared to AC, suggesting that dictamnine effectively inhibited goblet cell hyperplasia.

Masson’s trichrome (MT) staining showed marked peribronchial collagen deposition in the AC group (1944.40 ± 189.74 μm, *p* < 0.001) compared to the NC group (706.20 ± 23.51 μm). Dic 20 (1256.60 ± 249.82 μm, *p* < 0.05) reduced the fibrotic area compared to AC. These histological findings indicate that dictamnine improved abnormal tissue alterations in allergic asthma.

### 2.5. Dictamnine Modulated TGF-β/Smad Signaling and Tight Junction Protein Expression in the Lung Tissue of Asthmatic Mice

**Figure 5:**
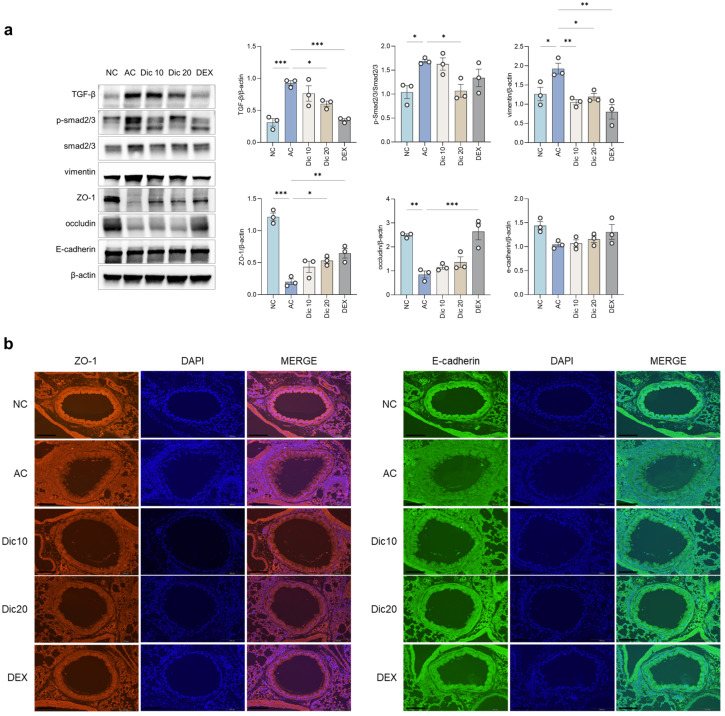
Dictamnine modulated TGF-β/Smad signaling and epithelial junction-related protein expression in OVA-induced asthma. (**a**) Representative Western blot images and quantitative analysis of protein expression in lung tissues: TGF-β, p-Smad2/3, ZO-1, vimentin, occludin, E-cadherin, and β-actin (loading control) in lung tissues from NC, AC, Dic 10, Dic 20, and DEX groups. (**b**) Representative immunofluorescence images of ZO-1 and E-cadherin with DAPI counterstaining (scale bar = 100 μm). Data are expressed as mean ± SEM (*n* = 3 per group). * *p* < 0.05, ** *p* < 0.01, and *** *p* < 0.001 compared to the AC group.

Dictamnine reduced the expression of TGF-β and p-Smad2/3 in lung tissues compared to the AC group (*p* < 0.05, *p* < 0.001, respectively), indicating suppression of TGF-β/Smad-related pathological signaling (Figure 5a). Vimentin expression was also decreased in the dictamnine-treated groups (*p* < 0.05, *p* < 0.01), supporting its anti-EMT effects. In contrast, ZO-1 expression was restored in Dic 20, suggesting improved epithelial barrier integrity (Figure 5a). Immunofluorescence staining (Figure 5b) indicated disrupted and discontinuous ZO-1 and E-cadherin staining in the AC group, whereas dictamnine restored a more continuous junctional pattern, particularly in the Dic 20 group.

Occludin and E-cadherin expression increased slightly, but the changes were not statistically significant. Together, these findings suggest that dictamnine ameliorated epithelial dysfunction by modulating TGF-β/Smad signaling and enhancing barrier stability.

### 2.6. Dictamnine Attenuated TGF-β/Smad Pathway Activation in Human Bronchial Epithelial (HBE) Cells

**Figure 6:**
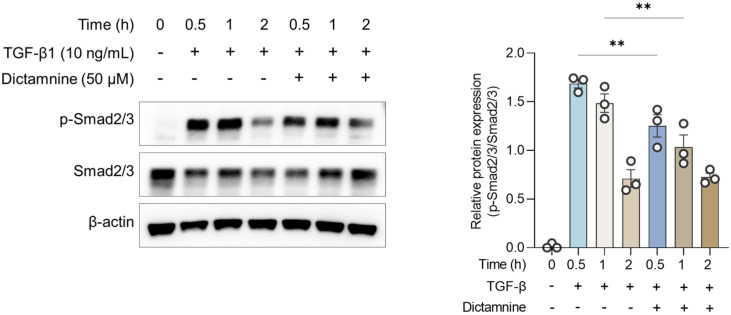
Dictamnine attenuated TGF-β-induced Smad2/3 phosphorylation in human bronchial epithelial (HBE) cells. Pretreatment with dictamnine (50 μM) reduced Smad2/3 phosphorylation at 0.5 and 1 h following TGF-β stimulation, as confirmed using Western blotting. Data are expressed as mean ± SEM (*n* = 3). ** *p* < 0.01 compared to the TGF-β-treated sample.

To further investigate the mechanisms through which dictamnine exerted its anti-asthmatic effects, its effect on TGF-β-induced responses was investigated in HBE cells. TGF-β-induced Smad2/3 phosphorylation substantially decreased post-dictamnine treatment, suggesting a direct inhibitory effect of TGF-β/Smad2/3 signaling (Figure 6).

### 2.7. Pathway Enrichment of Dictamnine-Associated Genes

**Figure 7:**
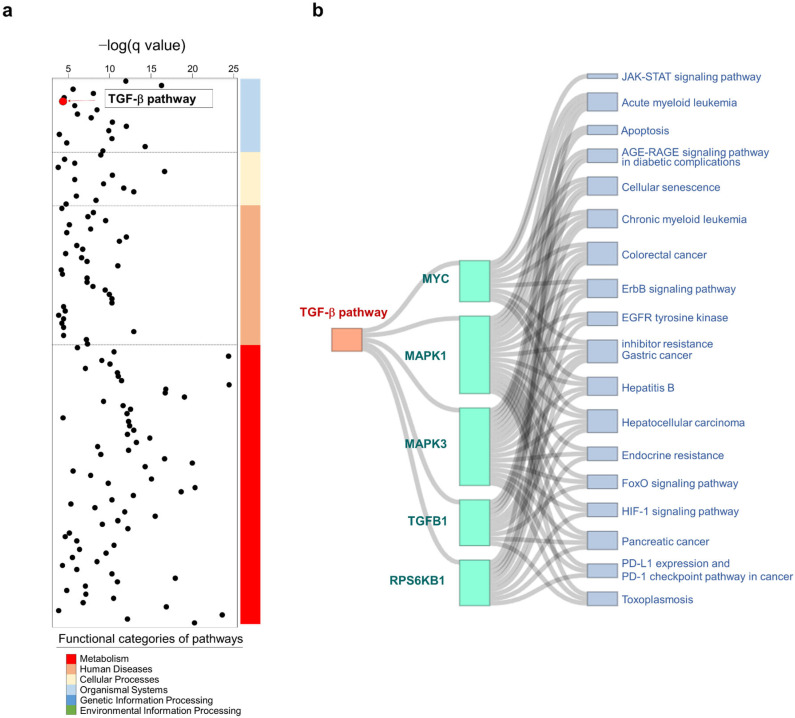
Network pharmacology analysis of dictamnine-associated genes. (**a**) Dot plot showing pathways enriched with dictamnine-related genes (*p* < 0.001, FDR < 0.001). Functional categories are color-coded and the TGF-β signaling pathway is highlighted. (**b**) Network representation of five dictamnine-related genes (*MYC*, *MAPK1*, *MAPK3*, *TGFB1*, and *RPS6KB1*) that are associated with the TGF-β pathways and several other signaling pathways.

A set of 45 genes potentially related to dictamnine action was identified from PubChem (Table S1). The enrichment analysis indicated that these genes were distributed across multiple biological pathways (*p* < 0.001, false discovery rate (FDR) < 0.001) (Figure 7a), and the TGF-β signaling pathway emerged as a relevant pathway. Five genes, *MYC*, *MAPK1*, *MAPK3*, *TGFB1*, and *RPS6KB1*, were mapped to the TGF-β pathway (Figure 7b). These genes were also involved in other signaling cascades, suggesting that dictamnine may exert broad biological functions, with the TGF-β pathway acting as one of its key regulatory axes.

## 3. Discussion

Although current asthma therapies effectively control airway inflammation and relieve symptoms, their use is still limited by concerns regarding adverse effects and the burden of long-term treatment [10,11,12]. Inhaled corticosteroids (ICS), the mainstay of asthma therapy, are associated with dose-dependent adverse effects, such as dysphonia, oropharyngeal candidiasis, impaired growth velocity in children, and reduced bone mineral density with chronic use [10,11]. Bronchodilators may cause tremor, palpitations, and cardiovascular concerns, and biological agents used for severe asthma can induce injection-site reactions and rare but serious hypersensitivity events [12]. Thus, identifying novel therapeutic agents that can effectively modulate airway inflammation is crucial to improve long-term asthma management. In the present study, dictamnine effectively mitigated airway inflammation in an OVA-induced asthmatic murine model. TGF-β and p-Smad2/3 expression and vimentin decreased post-dictamnine treatment, indicating that dictamnine acted by modulating the TGF-β/Smad signaling pathway and attenuated EMT.

Consistent with these in vivo findings, the in vitro experiments demonstrated that dictamnine substantially attenuated TGF-β-induced Smad2/3 phosphorylation. These results provide direct evidence that dictamnine interferes with TGF-β signaling at the epithelial cell level, supporting its role as a modulator of epithelial and inflammatory pathways. These findings suggest that modulating TGF-β/Smad signaling represents a promising strategy for reducing epithelial dysfunction and inflammation in asthma.

Dictamnine substantially reduced the MUC5AC level in BALF. PAS staining showed a marked decrease in the PAS-positive goblet cell count in the airway epithelium of dictamnine-treated mice, indicating suppression of goblet cell hyperplasia. These findings highlight the potential of dictamnine to effectively control mucus overproduction.

The AHR attenuation observed in this study may be partially explained by the suppression of Th2 cytokine expression, particularly IL-4 and IL-13, in BALF [19]. The concurrent decrease in MUC5AC expression and goblet cell hyperplasia support this mechanism. Upregulation of ZO-1 expression was observed after dictamnine treatment, implying partial restoration of epithelial barrier integrity. Although dictamnine did not substantially alter the expression levels of occludin and E-cadherin, they tended to increase in the treated groups.

At the molecular level, dictamnine modulated critical pathways associated with airway inflammation. Specifically, dictamnine suppressed TGF-β and p-Smad2/3 signaling, highlighting its potential to attenuate TGF-β-driven pathological changes, whereas it downregulated vimentin expression, suggesting its anti-EMT properties. The statistically significant upregulation of ZO-1 suggests that dictamnine may help maintain epithelial barrier integrity. These findings align with those of a previous study, which showed that TGF-β promotes fibrotic and structural airway changes by inducing extracellular matrix deposition and smooth muscle proliferation, contributing to airway wall thickening and fibrosis [20]. The ability of dictamnine to regulate these pathways highlights its potential to mitigate TGF-β-related pathogenic responses.

Tight junction proteins, such as ZO-1 and occludin, play crucial roles in maintaining airway epithelial barrier integrity. Disruption of the expression of these proteins increases epithelial permeability, facilitating the infiltration of allergen and inflammatory cells, which further exacerbates airway inflammation and barrier dysfunction through a TGF-β-driven mechanism [3]. The current study concurs; ZO-1 expression was substantially increased in Dic-treated lungs, indicating its potential to restore epithelial barrier function. Although immunofluorescence showed a mild recovery pattern in E-cadherin staining, this change was not statistically significant and thus requires validation in future studies.

Network pharmacology analysis identified *MYC*, *MAPC1/3*, *TGFB1*, and *RPS6KB1* as enriched genes in the TGF-β pathway, supporting the in vivo and in vitro results. Dictamnine may modulate multiple regulators associated with epithelial dysfunction inflammation.

This study has some limitations. An acute asthma model was used; therefore, the long-term, anti-asthmatic effects of Dic require validation in chronic asthma models. Future studies should also investigate the potential interactions with other signaling pathways involved in chronic asthma management. The TGF-β/Smad signaling pathway is linked with other pathways, such as the MAPK, STAT3, and β-catenin pathways, which influence airway remodeling and inflammatory responses [3,21]. Although the current study focused on TGF-β/Smad axis modulation, future research could elucidate whether Dic indirectly regulates these interconnected pathways to enhance its therapeutic efficacy. Particularly, further studies should experimentally validate the network pharmacology-predicted targets to clarify their roles in Dic-mediated protection against airway remodeling. Further mechanistic studies are required to determine the specific molecular steps at which Dic interacts with the TGF-β/Smad pathway. Further work using pharmacological and genetic approaches will be essential to substantiate the causal role of this signaling axis.

Dictamnine was administered orally in this study, whereas most clinical asthma therapies are delivered via inhalation to maximize pulmonary targeting and minimize systemic exposure. Oral administration was selected as it is a widely-used approach for early-stage screening of natural compounds. Future studies should explore whether inhalation-based delivery of Dic could improve lung-specific efficacy and therapeutic potential. Although basic pharmacokinetic data for dictamnine have been reported in healthy redolent [22], asthma-specific PK or exposure response information is still lacking. Thus, future studies should assess PK profiles and dose–response relationships in asthma models to determine the in vivo relevance of our findings.

## 4. Materials and Methods

### 4.1. Chemicals and Reagents

Dictamnine (≥98% purity) was procured from ChemFaces, Wuhan, China (CAS No. 484-29-7) and dexamethasone (positive control, DEX) from Sigma-Aldrich (St. Louis, MO, USA). Ovalbumin (OVA; Albumin from chicken egg white, Grade V; Sigma-Aldrich) and aluminum hydroxide (alhydrogel adjuvant 2%) were procured from InvivoGen (San Diego, CA, USA). The antibodies used for Western blotting were acquired from Cell Signaling Technology (Danvers, MA, USA) and Santa Cruz Biotechnology (Dallas, TX, USA). Catalogue numbers for all antibodies and ELISA kits are listed: TGF-β (#3711), p-Smad2/3 (#8828), Smad2/3 (#5678), ZO-1 (#5406), occludin (#91131), E-cadherin (#3195), vimentin (#5741), β-actin (#4970), anti-mouse secondary antibody (sc-516102), anti-rabbit secondary antibody (sc-2357), LEGENDplex Mouse Th cytokine panel (741044), Mouse MUC5AC ELISA kit (CSB-E11040m), LEGENDplex Mouse OVA-specific IgE kit (439807).

### 4.2. Animal Model and Ethical Approval

Female BALB/c mice (7-w old, 18–20 g; SLC, Shizuoka, Japan) were housed under specific pathogen-free conditions with a 12:12 h light/dark cycle with ad libitum access to food and water. All animal experiments were approved by the Institutional Animal Care and Use Committee of the Korean Institute of Oriental Medicine (KIOM-IAUCA-24-038) and conducted in accordance with the ARRIVE guidelines. Female BALB/c mice were used based on studies demonstrating their suitability as OVA-induced asthma models [23,24].

### 4.3. Experimental Design and OVA Sensitization/Challenge

Mice were assigned to five groups (*n* = 5/group) using stratified randomization based on body weight to ensure comparable baseline characteristics across groups: NC group, which was not subjected to OVA challenge; AC group, which underwent OVA sensitization and vehicle treatment; Dic-treated groups (Dic 10 and Dic 20), which underwent OVA sensitization with dictamnine at 10 and 20 mg/kg, respectively; and DEX group, which underwent OVA sensitization and dexamethasone treatment (1 mg/kg). The investigators who conducted outcome assessments were blinded to group allocation. Dictamnine doses were selected based on a study that demonstrated their efficacy and safety in a murine model [15]. Dexamethasone dose (1 mg/kg) was selected based on a study conducted on a mouse model of allergic airway inflammation [13]. The group size (*n* = 5 per group) was determined based on previous OVA-induced asthma studies demonstrating adequate statistical power [13].

Asthma was induced by sensitizing mice with 50 µg OVA emulsified in 2 mg aluminum hydroxide (alhydrogel adjuvant 2%, vac-alu-250; InvivoGen) administered intraperitoneally on days 0 and 7 (Figure 1a). Subsequently, the mice were challenged intranasally with 25 µg OVA in 50 µL PBS from days 14 to 17 under brief inhalation with isoflurane (2.5%) (TERRELL Isoflurane; Piramal Critical Care Inc., Bethlehem, PA, USA). Dictamnine (10 or 20 mg/kg) and dexamethasone (1 mg/kg) were orally administered once daily from days 11 to 17 at consistent times. Dictamnine and dexamethasone were suspended in sterile normal saline (Cleancle, JW Pharmaceutical Co., Seoul, Republic of Korea) and administered once daily by oral gavage. Mice in the NC and AC groups received the same volume of sterile normal saline by oral gavage under the same conditions.

### 4.4. AHR

AHR was assessed one day after the final OVA challenge using the FlexiVent system (SCIREQ Scientific Respiratory Equipment Inc., Montreal, QC, Canada). The mice were anesthetized with pentobarbital sodium (60 mg/kg, i.p.: Entobar; Hanlim Pharm Co., Seoul, Republic of Korea), a dose supported by previous murine asthma studies [25], and tracheostomized using an 18G metal cannula. Pancronium bromide (1 mg/kg, i.p.; Sigma-Aldrich, St. Louis, MO, USA) was administered to suppress spontaneous breathing. After securing mechanical ventilation, baseline Rrs was recorded. To evaluate bronchoconstriction, aerosolized methacholine (Sigma-Aldrich) was administered through a nebulizer at increasing concentrations (5, 10, 20, and 40 mg/mL). Each dose was administered for 3 min, followed by Rrs measurement. Data were processed and analyzed using FlexiVent (version 8.2.0; SCIREQ).

### 4.5. Sample Collection and Processing

On day 18, the mice were anesthetized using intraperitoneal injection of pentobarbital sodium (50 mg/kg; Hanlim Pharm Co.). The mice were euthanized and blood was sampled using abdominal vena cava puncture. The serum was separated using centrifugation (1800× *g*, 10 min) and stored at −80 °C until analysis.

BALF was obtained by lavaging the lung twice with 0.6 mL ice-cold PBS (total volume 1.2 mL). The BALF was centrifuged (160× *g*, 10 min, 4 °C), and the supernatants were stored at −70 °C for cytokine analysis.

After BALF collection, the lungs were harvested. The left lung was fixed in 10% neutral-buffered formalin for histological analysis and the right lung was snap-frozen in liquid nitrogen and stored at −80 °C for protein analysis.

### 4.6. Cytokine Analysis

Inflammatory cells were isolated using cytocentrifugation with a Cytospin device and stained with Diff-Quik (38721; Sysmex Co., Kobe, Japan). Differential cell counts were assessed to evaluate airway inflammation using a hemocytometer (C-chip, 2CH; NanoENTEK, Seoul, Republic of Korea) under a light microscope (×200) (BX53; Olympus, Tokyo, Japan), based on morphological and staining characteristics. Eosinophils were identified by their bilobed nuclei and reddish-orange cytoplasmic granules. Lymphocytes appeared as small round cells with dense nuclei and minimal cytoplasm. Macrophages displayed abundant pale cytoplasm and oval-shaped nuclei; neutrophils were recognized by their multilobed nuclei and lightly-stained granules.

BALF supernatants were analyzed for Th2 cytokine levels (IL-4, IL-5, and IL-13) using the LEGENDplex Mouse Th cytokine panel (741044; BioLegend, San Diego, CA, USA). MUC5AC level in BALF was determined using the Mouse MUC5AC ELISA kit (CSB-E11040m; Cusabio, Wuhan, China).

### 4.7. Serum IgE Analysis

To evaluate systemic allergic responses, blood samples collected after euthanasia were centrifuged at 1800× *g* for 10 min to obtain the serum, which was stored at −70 °C until further analysis. Serum OVA-specific IgE was quantified using the LEGEND MAX Mouse OVA-specific IgE ELISA kit (439807; BioLegend) as per the manufacturer’s instructions.

### 4.8. Histopathological Examination

Lung tissues were fixed in 10% neutral-buffered formalin and embedded in paraffin using an automatic tissue processor (Leica Microsystems, Wetzlar, Germany). The paraffin-embedded lung sections (4 μm) were stained with hematoxylin and eosin to assess airway inflammation, PAS to evaluate goblet cell hyperplasia and mucus production, and MT to evaluate pulmonary fibrosis. The stained sections were scanned using a digital slide scanning system (KFBIO Technology for Health, Ningbo, China), and images were captured with the MoticsSlide Scanner (VM1) and Motic VM3.0 software (Motic, Xiamen, China). All histological analyses were performed under a light microscope at ×200 unless specified otherwise.

Airway inflammation was scored based on the severity of peribronchial inflammatory infiltration (0 = none, 1 = mild, 2 = moderate, 3 = severe). Goblet cell hyperplasia was scored based on the percentage of PAS-positive epithelial cells (1 = <25%, 2 = 25–50%, 3 = 50–75%, 4 = >75%). Fibrosis was assessed by calculating the MT-positive area using image analysis software (VM3.0; Motic, Xiamen, China).

### 4.9. Immunofluorescence Staining

The lung tissue sections were deparaffinized and rehydrated through a graded ethanol series. After blocking with 5% BSA for 1 h at 25 °C, the sections were incubated overnight at 4 °C with primary antibodies: zonula occludens-1 (ZO-1, 1:100, #NBPa-85047; Novus Biologicals, Centennial, CO, USA) and E-cadherin (1:100, #3195; Cell Signaling Technology). Following three washes with PBS, the sections were incubated with Alexa Fluor-conjugated secondary antibodies (goat anti-rabbit, 1:1000, Alexa Fluor 594 or Alexa Fluor 488; Thermo Fisher Scientific, Waltham, MA, USA) for 60 min at 25 °C in the dark. The nuclei were counterstained with DAPI using Antifade Mounting Medium with DAPI (Vector Laboratories, Burlingame, CA, USA). Fluorescence images were captured using a fluorescence microscope (BX53; Olympus).

### 4.10. Cell Culture and Treatment

Primary HBE cells (PCS-300-100; ATCC, Manassas, VA, USA) were cultured in airway epithelial cell basal medium (PCS-300-030; ATCC) supplemented with the bronchial epithelial cell growth kit (PCS-300-040; ATCC) under standard conditions (37 °C, 5% humidified atmosphere). Cells at passages 3–7 were used in all experiments.

The cells were pretreated with Dic (50 μM) for 1 h after replacing the culture medium with basal medium and then stimulated with or without recombinant human TGF-β (10 ng/mL) for 0.5, 1, or 2 h.

### 4.11. Western Blotting

Lung samples were homogenized using a Precellys-24 homogenizer (Bertin Technologies, Montigny-le-Bretonneux, France) with Hard Tissue Homogenizing CK28-R beads (Bertin Technologies) in RIPA buffer supplemented with a protease inhibitor cocktail (LPS Solutions, Daejeon, Republic of Korea). Protein concentration was determined using a Pierce BCA Protein Assay Kit (Thermo Fisher Scientific), and 20 μg of protein per lane was separated on 10% Mini-PROTEAN TGX gels (Bio-Rad Laboratories, Hercules, CA, USA) using sodium dodecyl sulphate–polyacrylamide gel electrophoresis. Proteins were then transferred onto polyvinylidene fluoride membranes (Pall GmBH, Dreieich, Germany). After blocking with 5% BSA at 25 °C for 1 h, the membranes were incubated overnight at 4 °C with primary antibodies targeting TGF-β (1:1000; #3711), p-Smad2/3 (1:1000; #8828), Smad2/3 (1:1000; #5678), ZO-1 (1:1000; #5406), occludin (1:1000; #91131), E-cadherin (1:1000; #3195), vimentin (1:1000; #5741), and β-actin (1:2000; #4970) from Cell Signaling Technology. After washing with Tris-buffered saline containing 0.1% Tween-20, the membranes were incubated with HRP-conjugated secondary antibodies (goat anti-mouse, 1:2000; sc-516102 or goat anti-rabbit, 1:2000, sc-2357; Santa Cruz Biotechnology) for 1 h at 25 °C. Protein bands were visualized using the Super Signal West Femto Maximum Sensitivity Substrate (Thermo Fisher Scientific) and detected with a ChemiDoc Imaging System (Bio-Rad Laboratories). The intensity of the protein bands was quantified using Image Lab Software version 5.2.1 (Bio-Rad Laboratories).

### 4.12. Network Pharmacology Analysis

Potential Dic-associated targets were retrieved from the PubChem database (CID: 68085; http://pubchem.ncbi.nln.nih.gov, accessed on 10 December 2025). Pathway enrichment analysis was conducted using the DAVID platform (http://david.ncifcrf.gov, accessed on 10 December 2025), with default statistical settings comprising the modified Fisher’s exact test and Benjamini–Hochberg FDR adjustment [Dennis 2003 [26]]. Information on individual pathways and their functional categories was obtained from Kyoto Encyclopedia of Genes and Genomes (KEGG) (http://www.genome.jp/kegg, accessed on 10 December 2025).

### 4.13. Data and Statistical Analysis

All data are presented as mean ± standard error of the mean, with *n* values specified in the figure legends. Group sizes (*n* = 5 per group) were determined based on previous studies using the OVA-induced asthma model [13]. Statistical comparisons were conducted using one-way analysis of variance, followed by Dunnett’s multiple comparisons test or two-way analysis of variance with Bonferroni’s post-hoc adjustment. The analyses were conducted using GraphPad Prism software (version 9.5.1; GraphPad Software, San Diego, CA, USA). Statistical significance was set at *p* < 0.05.

## 5. Conclusions

Dictamnine exerts therapeutic effects in asthma by simultaneously attenuating airway inflammation and remodeling. It suppresses TGF-β/Smad2/3 signaling in lung tissue and bronchial epithelial cells, downregulates vimentin expression, and restores ZO-1 expression, suggesting anti-EMT and barrier-protective actions. Network pharmacology analysis supported the involvement of TGF-β-related genes. Collectively, Dic shows promise as a therapeutic agent for asthma.

## Data Availability

The original contributions presented in this study are included in the article/Supplementary Materials. Further inquiries can be directed to the corresponding author.

## Supplementary Materials

The following supporting information can be downloaded at: https://www.mdpi.com/article/10.3390/ijms262411891/s1.

## Abbreviations

AHR	airway hyperresponsiveness
EMT	epithelial–mesenchymal transition
MUC5AC	mucin 5AC
HIF	hypoxia-inducible factor
OVA	ovalbumin
AC	asthma control
NC	normal control
Rrs	airway resistance
BALF	bronchoalveolar lavage fluid
Dic	dictamnine
PAS	Periodic Acid–Schiff
MT	Masson’s Trichrome
HBE	human bronchial epithelial
FDR	false discovery rate
