# Supplementary material for: Dictamnine Exhibits Anti-Asthmatic Effects by Modulating TGF-β/Smad2/3 Signaling in a Murine Asthma Model and Human Bronchial Epithelial Cells
Source: Int J Mol Sci. 2025 Dec 10;26(24):11891. doi: 10.3390/ijms262411891 (PMC12733116; doi:10.3390/ijms262411891)
Supplement: Supplementary file 1 [file ijms-26-11891-s001.zip › ijms-3986903-supplementary.pdf]

Table S1. List of dictamnine-related genes retrieved from PubChem (http://pubchem.ncbi.nlm.nih.gov).

No.	Gene Symbol	Entrez ID
1	*AKT1*	207
2	*BAG3*	9531
3	*BAX*	581
4	*BBC3*	27113
5	*BCL2*	596
6	*BCL2L1*	598
7	*BID*	637
8	*BRAF*	673
9	*CASP3*	836
10	*CD44*	960
11	*CDH1*	999
12	*CDH2*	1000
13	*CDK1*	983
14	*CTNNB1*	1499
15	*EIF4E*	1977
16	*EIF4EBP1*	1978
17	*FGF*	582058
18	*GSK3B*	2932
19	*HGF*	3082
20	*HIF1A*	3091
21	*IL1B*	3553
22	*IL6*	3569
23	*JUN*	3725
24	*MAPK1*	5594
25	*MAPK14*	1432
26	*MAPK3*	5595
27	*MAPK8*	5599
28	*MCL1*	4170
29	*MET*	4233
30	*MKI67*	4288
31	*MMP2*	4313
32	*MMP9*	4318
33	*MTOR*	2475
34	*MYC*	4609
35	*OCLN*	100506658
36	*PARP1*	142
37	*PTK2*	5747
38	*PXN*	5829
39	*RAF1*	5894
40	*RPS6KB1*	6198
41	*SNAI2*	6591
42	*STAT3*	6774
43	*TGFB1*	7040
44	*TP53*	7157
45	*VIM*	7431
